# Laxative Pill

**Published:** 1877-01-20

**Authors:** 


*Laxative Pill—*

R. Aloes.....................two scruples,
Nux vomica..............five grains.
Ft. pills No. xx. Dose i pill.

